# An unusual cause of a breast mass in a 13-year-old girl: a case report

**DOI:** 10.1186/s13256-018-1761-5

**Published:** 2018-08-30

**Authors:** Wafaa Ghazali, Kholoud Awagi, Ghadah AlZahrani, Laila Ashkar, Zuhoor AlGaithy

**Affiliations:** 1 Department of Surgery, University of Jeddah, Jeddah, Saudi Arabia; 2 Department of Surgery, King Abdulaziz University, Jeddah, Saudi Arabia; 3 Department of Radiology, King Abdulaziz University, Jeddah, Saudi Arabia

**Keywords:** Breast mass, Foreign body, Retained temporary epicardial pacing wire

## Abstract

**Background:**

Adolescents rarely present with breast lumps, and such lumps are usually due to benign causes. Foreign bodies in the breast are an uncommon finding and could be detected incidentally during imaging or be symptomatic and present as a painful mass. Sometimes they cause diagnostic dilemmas as they mimic malignancies. To the best of our knowledge, this is the second case reported in the literature about an abscess caused by a migrating retained temporary epicardial pacing wire.

**Case presentation:**

A 13-year-old girl of African ancestry was referred to our clinic with a left breast mass that had been gradually increasing in size for 2 years. The mass was tender but was not associated with skin changes, nipple discharge, or fever. She had a history of rheumatic heart disease and had undergone mitral and tricuspid valve repair more than 2 years ago. Blood work and biochemistry were within normal ranges. An ultrasound of her left breast showed a large, irregular, complex, heterogeneous mass measuring 4.3 × 2.7 × 3.5 cm at 6 o’clock position with central cystic changes but no significant intrinsic vascular flow. There was significant associated skin and subcutaneous edema. Given the echogenicity of the mass, an infectious cause was considered likely, and malignancy was less likely but could not be excluded. An ultrasound-guided biopsy was performed and revealed cores of breast tissue heavily infiltrated with mixed acute and chronic inflammatory cells, consistent with a chronic abscess. She received a 10-day course of antibiotics. However, she remained symptomatic, and the mass did not decrease in size. Therefore, we proceeded to surgical excision. The breast mass was excised. It was fixed to the underlying rib, and a thin, long, metallic wire that moved with her heartbeat was observed protruding from a small opening above the rib. This was a migrated retained epicardial pacing wire from the previous valve repair surgery. The histopathology of the mass revealed mammary tissue with acute and chronic inflammatory cells.

**Conclusion:**

Temporary epicardial pacing wires should be removed completely by cardiothoracic surgeons after surgery to avoid migration that might lead to unexpected complications.

## Background

Breast lumps in children and adolescents are usually benign. Fibroadenoma is the most common cause of such lumps; abscesses and inflammatory causes are rare [1]. Malignancy in this age group is extremely rare. The most common malignancy is phyllodes tumor [2]. Breast masses due to foreign bodies are infrequent findings. We report here a case of a 13-year-old girl who presented with a left breast mass secondary to a retained temporary epicardial pacing wire (TEPW).

## Case presentation

A 13-year-old girl of African ancestry was referred to our breast clinic for evaluation of a left breast mass. She had been complaining of the left breast lump for 2 years. The lump was gradually increasing in size and it was tender. There was no history of skin changes, nipple discharge, fever, or trauma. Furthermore, there was no family history of similar conditions, no history of traveling abroad, and no contact with a person with tuberculosis. Her medical history revealed history of rheumatic heart disease. She underwent mitral and tricuspid valve repair more than 2 years prior to presentation at our breast clinic. She was a student in primary school living with her parents and siblings.

**Fig. 1:**
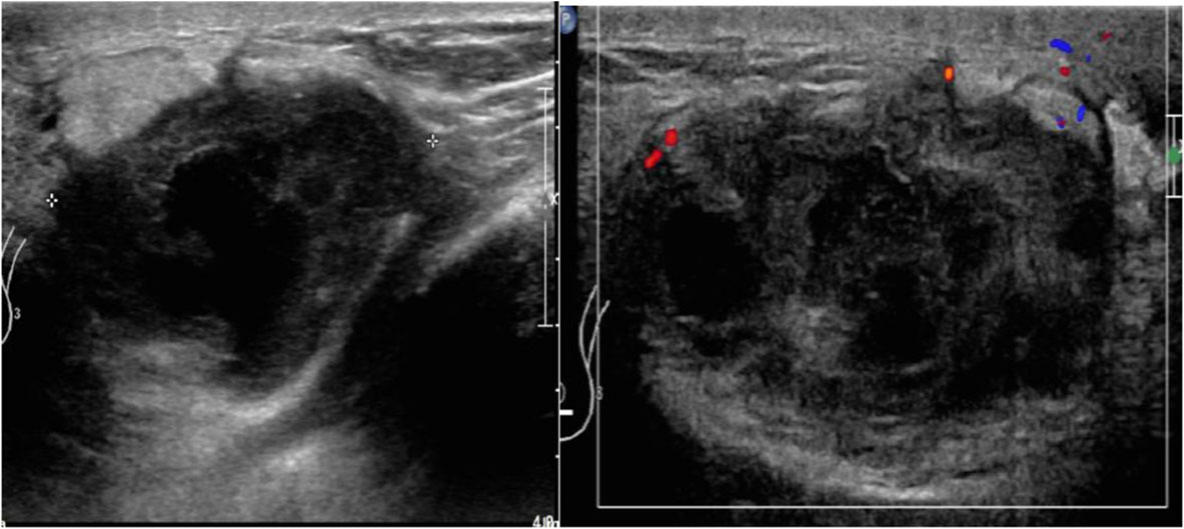
Ultrasound of the left breast

On examination she was hemodynamically stable. She had a normal body build for her age. She was not pale or jaundiced. A breast examination revealed an irregular left breast mass that was palpable at six o’clock position. The mass was approximately 4 cm in maximal diameter; it was hard, tender, and fixed on the posteromedial side. There were no inflammatory skin changes or any nipple changes. Her right breast was unremarkable. There were no palpable bilateral axillary lymph nodes. Abdomen, chest, and neurological examinations were unremarkable. Her blood work, including complete blood count, liver function test, urea and electrolytes, and coagulation profile, was within normal ranges. Ultrasound of her left breast (Fig. 1) showed a large, irregular, complex, heterogeneous mass measuring 4.3 × 2.7 × 3.5 cm at 6 o’clock position. There were central cystic changes but no significant intrinsic vascular flow. There was significant associated skin and subcutaneous edema and thickening with fluid seen tracking within subcutaneous tissue. The surrounding fat appeared more echogenic, consistent with the inflammatory and infectious changes seen in breast abscesses. Given the echogenicity of the mass, an infectious cause was suspected and malignancy was less likely but could not be excluded. An ultrasound-guided biopsy was recommended. A left axillary lymph node appeared prominent with a cortical thickness of 5 mm.

An ultrasound-guided aspiration of the cystic portion was attempted with an 18-gauge needle. Minimal yellowish fluid was retrieved and sent for aerobic bacteria, anaerobic bacteria, and fungi culture and sensitivity analysis. A biopsy of the mass was performed. The culture showed moderate growth of *Staphylococcus aureus*. An acid-fast bacilli stain was negative. Microscopic *tuberculosis* bacilli were not detected. Histopathology sections revealed cores of breast tissue heavily infiltrated with mixed acute and chronic inflammatory cells. The diagnosis was consistent with chronic abscess. She received one gram of amoxicillin and clavulanate potassium every 12 hours for 10 days. However, she remained symptomatic, and the mass did not decrease in size. Therefore, we proceeded to surgical excision.

**Fig. 2:**
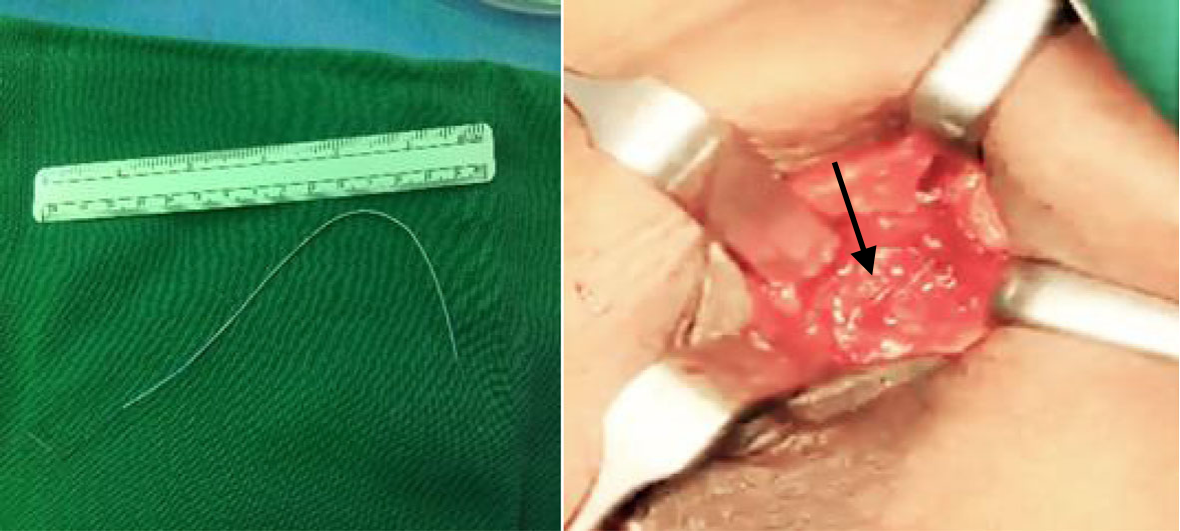
The pacemaker wire (arrow)

Excision of the breast lump through a peri-areolar incision was performed. During the operation, we found a circumscribed semi-cystic lesion that was not typical of fibroadenoma; the mass was fixed to the underlying rib. The cystic part was opened, and the turbid fluid that emerged was sent for culture. A long, thin, metallic wire was found inside the cavity; it emerged from a small opening above the rib and moved synchronously with her heartbeat (Fig. 2).

A cardiothoracic surgeon was consulted intraoperatively. The wire was removed; it was a retained TEPW that was inserted during the tricuspid and mitral valve repair procedure and had migrated to the breast. The mass was excised and sent for histopathology, which revealed mammary tissue with acute and chronic inflammatory cells.

She was discharged to home on the same day and was followed up at our out-patient clinic 2 weeks later. Her wound site was clean and dry. There were no palpable masses and no signs of inflammation. She was later seen at our out-patient clinic for a 1-year post-surgery follow-up. She had no complaints, and a breast examination revealed no palpable masses.

## Discussion

Breast masses are uncommon in adolescents; of young girls, 3.2% present with a breast lump [3]. The masses are usually due to benign causes. Fibroadenoma is the most common cause (30–40%), while abscesses and inflammatory causes are uncommon (0–7%) [4]. Malignant breast masses in this population are very rare; such cases are more commonly metastatic cancer to the breast than primary breast cancer. Rhabdomyosarcoma, neuroblastoma, lymphoma, and leukemia are known malignancies that metastasize to the breast [2, 4].

Proper history and physical examination are essential steps to assess an adolescent with a breast lump. Diagnostic imaging is very important for complete assessment. Ultrasound is a key diagnostic tool in adolescents as it provides data on the lump characteristics (benign or malignant); moreover, ultrasound is a safe modality that does not involve any radiation that may harm children [2].

Breast abscesses are associated with a history of piercing, trauma, lactation, and previous breast surgery. The patient presents with a painful breast mass associated with redness, tenderness, fever, and increasing white cell count. Ultrasound helps confirm the diagnosis and guides drainage. Patients usually respond to a course of antibiotics [5].

Most breast lesions in adolescents are managed conservatively with reassurance and clinical follow-up; they are usually self-limited [5]. Some cases require surgical intervention when imaging cannot exclude malignancy or in cases of biopsy-proven malignancy, growing masses, or the absence of clinical improvement [6].

Here we report an unusual cause of a breast lump secondary to a migrated TEPW in a 13-year-old girl with a history of rheumatic heart disease, for which she underwent mitral and tricuspid valve surgery.

In our case, we chose surgical intervention because the mass did not decrease in size after drainage and antibiotic treatment. In addition, ultrasound showed a complex mass that was not drained completely, and the radiologist labeled it as Breast Imaging Reporting and Data System (BIRADS) 4.

Foreign bodies in the breast are not commonly reported in the literature. They are detected incidentally during mammogram or ultrasound or present as breast masses mimicking carcinoma or breast abscesses. Foreign bodies reported in the literature include retained silicone drain, suture material, bullets, and migrated wires, such as those for localization [7].

Jafferjee and her colleagues reported the case of a breast abscess caused by a migrating TEPW in a 60-year-old woman with multiple comorbidities presenting 7 years after mitral and tricuspid valve repair [8]. These wires are routinely used in open-heart surgery as diagnostic and therapeutic tools to detect and treat cardiac arrhythmias during the postoperative period. They are sutured to the ventricle or atrium during surgery and are usually removed by gentle traction or cut flush with the skin. The residual wire is expected to retract into the tissue before discharging the patient [9]. Retained pacing wires cause no symptoms in most cases; however, they have been related to some complications reported in the literature including hemopericardium [10], bronchocutaneous fistula [11], retroaortic abscess [12], prosthetic valve endocarditis [13], and migration to the intraperitoneal cavity [14] and breast [8]. Our patient underwent tricuspid valve repair 2 years prior to presentation. The TEPW was not removed, and it migrated to her left breast where it served as a nest for infection. Consequently, the retained TEPW led to a foreign body reaction, causing the formation and progression of a chronic breast abscess that presented as a breast lump creating a diagnostic dilemma.

## Conclusions

The goal of this case report was to promote awareness among the medical community about this rare complication of cardiac pacemaker placement and to illustrate the importance of including migrating foreign body in the differential diagnosis of breast lesions with clinical and radiological inflammatory features. In addition, we believe that a cardiothoracic surgeon should completely remove TEPW soon after cardiac surgery to avoid migration that might lead to unexpected complications.

## Abbreviation

TEPW: Temporary epicardial pacing wire
